# Face Mask Wastes as Cementitious Materials: A Possible Solution to a Big Concern

**DOI:** 10.3390/ma15041371

**Published:** 2022-02-12

**Authors:** Marta Castellote, Eva Jiménez-Relinque, María Grande, Francisco J. Rubiano, Ángel Castillo

**Affiliations:** Institute of Construction Science Eduardo Torroja (IETcc-CSIC), 28050 Madrid, Spain; eva.jimenez@csic.es (E.J.-R.); m.grande@ietcc.csic.es (M.G.); franrs12@ietcc.csic.es (F.J.R.); a.castillo@ietcc.csic.es (Á.C.)

**Keywords:** face masks, addition to mortars, circular economy, strength and durability properties

## Abstract

After more than two years wearing surgical masks due to the COVID-19 pandemic, used masks have become a significant risk for ecosystems, as they are producing wastes in huge amounts. They are a potential source of disturbance by themselves and as microplastic contamination in the water system. As 5500 tons of face masks are estimated to be used each year, there is an urgent need to manage them according to the circular economy principles and avoid their inadequate disposal. In this paper, surgical wear masks (WM), without any further pretreatment, have been introduced as addition to mortars up to 5% in the weight of cement. Mechanical and microstructural characterization have been carried out. The results indicate that adding MW to the cement supposes a decrease in the properties of the material, concerning both strength and durability behavior. However, even adding a 5% of WM in weight of cement, the aspect of the mortars is quite good, the flexural strength is not significantly affected, and the strength and durability parameters are maintained at levels that—even lower than the reference—are quite reasonable for use. Provided that the worldwide production of cement is around 4.1 Bt/year, the introduction of a 5% of WM in less than 1% of the cement produced, would make it possible to get rid of the mask waste being produced.

## 1. Introduction

Due to the COVID-19 pandemic, people have started wearing surgical masks in order to take precautionary measures, which has dramatically increased the amount of waste created [1,2]. According to [3], one surgical mask per person, per day for a year in the UK would create over 124,000 tons of plastic waste. WHO estimated that nearly 89 million masks were needed to control COVID-19 each month [4]. Face masks are a source of microplastic contaminants in water ecosystems [5,6,7,8,9] and in indoor and outdoor air [10,11], as polypropylene and other plastics—polystyrene, polycarbonate, polyethylene, or polyester, among others—are used in making face masks. This constitutes a big problem related to health for different living beings, including humans and the environment as a whole [12]. Thus, some voices are claiming that the circular economy principle should guide policy making for the management of medical waste and, specifically, single-use face masks [13,14,15,16,17,18,19]. A life cycle analysis of single-use and reusable face masks can be found in [20].

Since the beginning of the pandemic, several researchers have made a characterization of face masks using several techniques. Mainly, the studies have been focused on disinfection and reuse of the masks, among other solutions [21,22,23,24,25]. In [26], the mask were characterized by thermal, morphological, and chemical analyses, proposing a recycling of the resulting material after thermal treatment. In [27], the pore structure of the surgical mask was investigated after treatment with ethyl alcohol, UV light, steam, or a washing machine. Peinador et al. [28] used capillary flow and liquid extrusion porometry to characterize pore size distributions. Scarce studies have arisen concerning recycling used masks. Crespo el al. [29] demonstrated the possibility of recycling face masks using the same protocols that are used in mechanical recycling of thermoplastics. In [30], the masks were transformed into S-doped porous carbon as the cathode electrode for supercapacitors. In [31], researchers demonstrated that, depending on the type of mask, the sound pressure level transmitted is different; additionally, [32] presented the results of an experimental study on the recycled material obtained from masks, including characterization as bulk density, fiber diameter, porosity, flow resistivity, and tortuosity, as well as acoustic efficiency. In [33], the polypropylene fibres from the masks were blended with acrylonitrile butadiene rubber. In [34], researchers presented a theoretical strategy of disposing the masks by their conversion to alternative fuel. In [35], shredded face masks were added to recycled concrete aggregate (RCA) for road base and subbase applications.

The use of polypropylene fibers has been previously described and extensively used in the concrete sector, mainly due to their high tensile strength and Young’s modulus, but also due to improvement of some properties, such as shrinkage and high alkaline resistance [36,37,38,39,40,41]; that is why face mask wastes are good candidates to be reused in cement-based materials. Rehman el al. [42] added shredded face masks and silica fume to cement to stabilize fat clay soils. The only paper found using face masks as an addition to produce cementitious materials is [43], where the masks were introduced by volume of concrete up to 0.25%. According to the mixes given in [43], the 0.25% in volume of concrete corresponds to 0.37% and 2.2% in weight of concrete and cement, respectively. These percentages seem to be quite low for dealing with the huge environmental problem of mask waste. In addition, the focus of the paper was put on the mechanical properties of the resulting mixes, not paying attention to aspects of durability. 

In this paper, surgical wear masks without any pretreatment were introduced as addition to mortars up to 5% in the weight of the cement. Mechanical and microstructural characterization was carried out.

## 2. Materials and Methods

### 2.1. Materials and Samples Preparation

**Figure 1:**
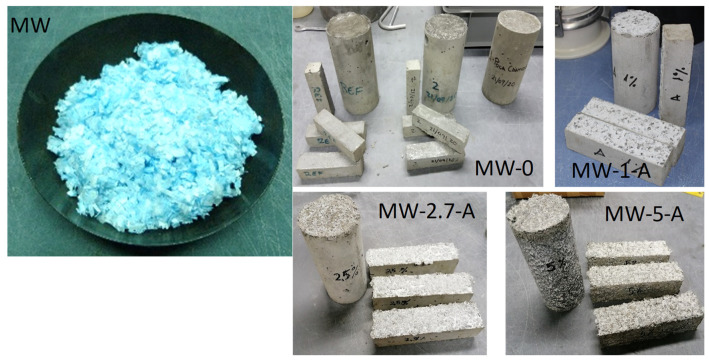
Shredded MW ready to be included in the mortar, and aspect of some of the mixes.

Surgical masks were used in this study. The metal nose-fitting piece and the elastic ear bands were manually cut with scissors and discarded, and the filtering parts of the masks were cut into small pieces by means of a paper shredder, passed through in both directions, obtaining small squares. The resulting aspect is given in Figure 1. This material has been labelled as MW (mask waste).

Mortars were cast according to the compositions given in Table 1, with a water/cement ratio of 0.4, the same amount of cement as sand, and different amounts of MW, ranging from the reference mix to 5% in the weight of the cement. The samples labelled as “A” were cast adding the dry MW at the same time as the sand. For the mix with 1% MW, for the sample labelled “B”, 2/3 of the additive was mixed with the MW before incorporating it to the mix. 

The mixing procedure was carried out following the standard UNE-EN-196-1. Mortars were cast in two types of molds—40 × 40 × 160 mm blocks and 75 mm ϕ × 150 mm cylinders—and were cured in a humid chamber for 28 days. The aspect of the different mixes can be seen in Figure 1.

### 2.2. Experimental Program

Compressive and flexural tensile strengths of the mortars were determined according to the UNE-EN 1015-11 standard. The two residual portions of the prisms after the flexural strength test were used for the compressive strength test. All tests were carried out on duplicate specimens. Samples were characterized through back scattering electron microscopy (BSE, Hitachi S-4800 microscope Tokio, Japan) with microanalysis (EDX, Bruker Corporation, Ettlingen, Germany), mercury intrusion porosimetry (MIP, Autopore IV model 9505 by Micromeritics Instrument Corporation, Norcross, GA, USA), resistivity, and capillary absorption. After more than 100 days in the humid chamber for every sample, they were submitted to a heating test, following the slope given by ISO 834 standard, up to 1024 °C in 90 min, with this temperature maintained for 1 h. After 24 h in the oven, turned off, they were submitted to other compressive strength tests. For the samples with higher amounts of MW (1%, 2.7%, and 5%), the effective (D_eff_) and apparent (D_app_) chloride diffusion coefficients were determined through the multiregime method [44].

## 3. Results and Discussion

**Figure 2:**
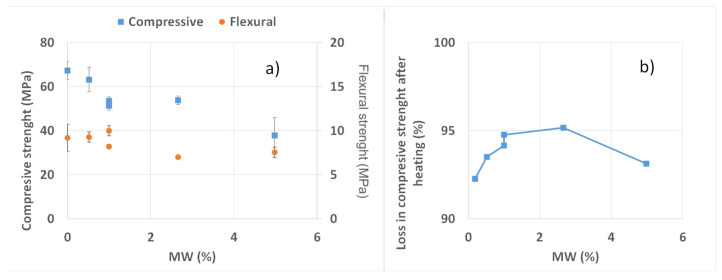
(**a**) Compressive and flexural strength for the samples with different MW (%). The standard deviations are given as error bars. (**b**) Loss in compressive strength after the heating tests with respect to the initial values.

Figure 2a,b summarizes the results of mechanical strength. Figure 2a shows that, as the amount of MW increases, the greater the decrease in compressive strength, indicating around a 6% decrease for 0.5% MW, around a 20% decrease in the range 1–3% MW, and a higher diminution (44%) for a 5% MW. This is not in agreement with [43], where an increase in compressive strength was observed until reaching a mask content in volume of 2%, equivalent to 1.7% by the weight of the cement. Concerning flexural strength, the increase in MW does not imply significant changes. The samples containing 0.5% and 1% (B) even increased their value with respect to the reference sample, having a 9% higher flexural strength than the MW-1-B sample. Concerning the method of including the MW, for both parameters, sample B gave higher values. 

Figure 2b shows the decrease in the compressive strength for the different samples after heating the specimens until 1024ºC. It can be seen that all the samples present a loss of strength higher than 90%, increasing to around 2% of MW, and decreasing thereafter, for the MW-5-A sample.

**Figure 3:**
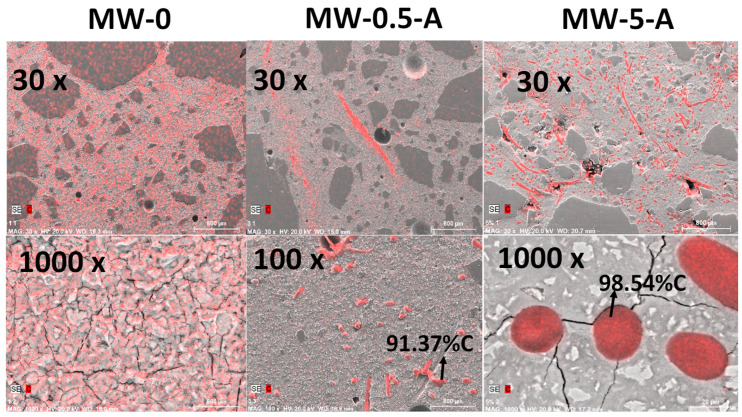
Elemental mapping images for carbon (in red) taken by BSE on golden metalized samples for different amounts of MW in the mortars.

Figure 3 presents the elemental mapping images for carbon (in red) taken by BSE on golden metalized samples for different amounts of MW in the mortars (reference, 0.5, and 5%). At 30 magnifications, all the samples present good aspect, with some air pores in all of them and they were well compacted. At 1000 magnifications, the reference exhibited microcracks all around the mortar. The pieces of masks can be seen as spots (perpendicular to the surface) or fibers, all of them having a percentage of C higher than 90%. They are distributed quite homogeneously in the mortars, not agglomerated but as isolated pieces. Comparing the reference and the MW-5-A mortars at 1000 magnifications, it can be seen that the network of microcracks present in MW-0 disappeared; finding the microcracks radially distributed from the MW portions. The interface between the MW and the cement paste seems to be tight, without appreciable interfacial weakest zones. 

**Figure 4:**
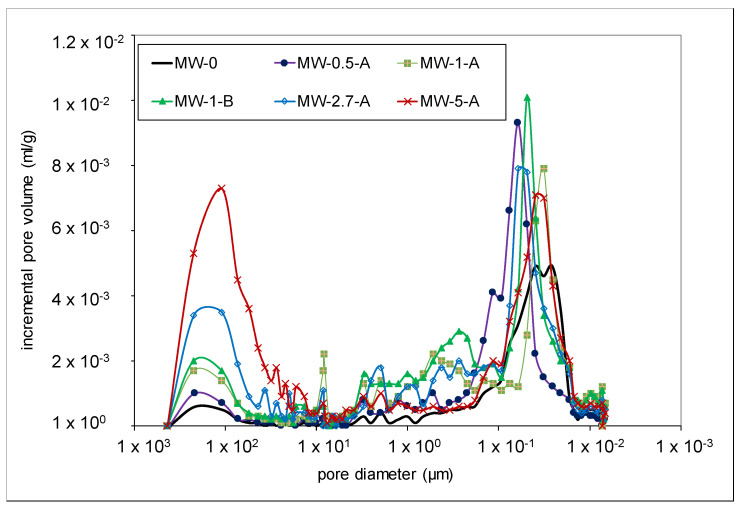
Differential pore size distributions of the specimens.

Table 2 shows the results from mercury intrusion porosimetry, in which the total porosity (percent in volume), the mean pore diameter, and the bulk density of the hardened samples are given. A more detailed analysis can be performed by observing the differential pore size distribution, as shown in Figure 4. 

From the results in Table 2 and Figure 4, it can be realized that the most significant changes are produced in the air porosity, with an incremental increase in the pores bigger than 100 µm as the amount of MW increased. In addition, there is an increase and a shift in the maximum corresponding to the smallest pores towards higher dimeters. However, it is remarkable that, unlike the progressive increase in the bigger pores, the porosity and the average pore diameter stabilized at around 1% of MW, which implies a higher refinement in the small pores at higher values of MW added, as can be realized in Figure 4. 

**Figure 5:**
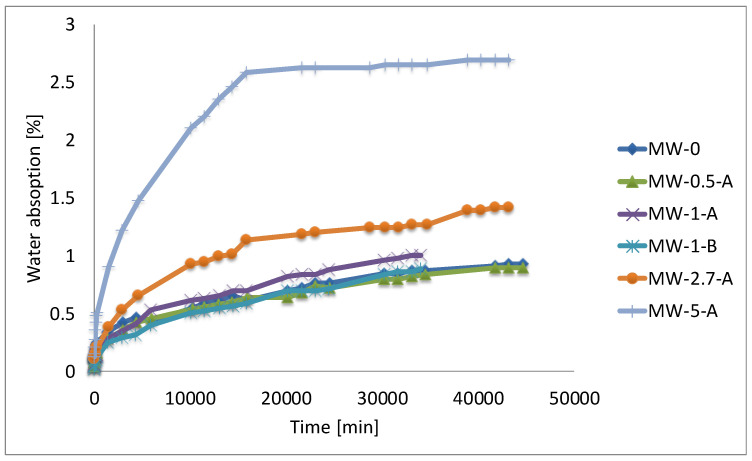
Water absorption for the different samples.

Concerning capillary absorption, the percentage of increase in weight due to the uptake of water in specimens of 75 mm diameter and 50 mm length is given in Figure 5, where it can be seen that, as the amount of WM increases, the uptake of water increases.

**Figure 6:**
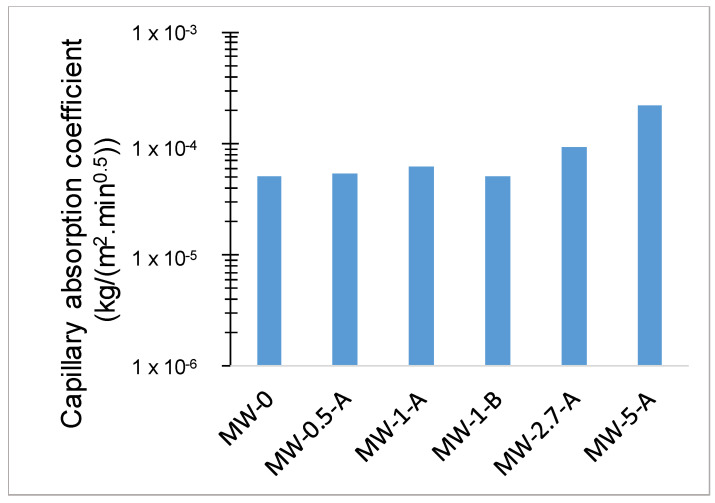
Capillary absorption coefficient for the different samples.

The capillary absorption coefficient for the different samples is given in Figure 6, where it can be seen that increasing WM above 1% makes the capillary adsorption coefficient increases, and, correspondingly, the resistance of the sample to the uptake of water decreases. 

**Figure 7:**
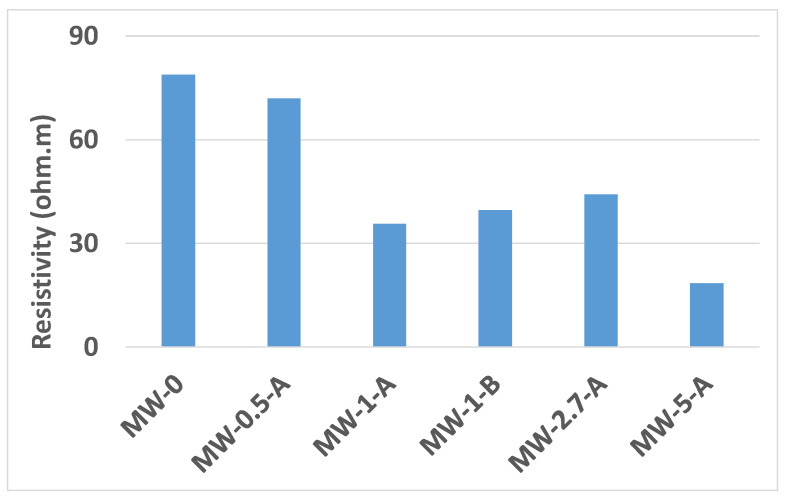
Resistivity of the different samples.

The resistivity of the saturated samples, after 28 days curing, is given in Figure 7, where it can be seen that the reference presents the highest resistivity, in agreement with the results of the rest of durability parameters. The chloride effective diffusion coefficient has been calculated from the resistivity value according to [45], and both coefficients (D_eff_ and D_app_) have been determined for the highest amounts of MW. The results are presented in Table 3, where it can be seen that for the samples including MW, the values calculated from resistivity are higher than those experimentally determined.

The guide—“Concrete design for a given structure service life”—by the Association Française de Génie Civil (AFGC) [46], gives the indicative classes and limit values relating durability indicators for concrete. Even though the materials tested here are mortars, they can give a reference on the quality of the materials. The classification of the mortars with MW, obtained in this research according to the thresholds given in [46], is given in Table 4.

It has to be noticed that the limit values defining the durability classes in Table 4 correspond to measurements performed on concrete and in accordance with the methods described in the guide [46], and on test specimens water-cured for three months after casting. In the case of the mixes tested here, the samples are mortars and were cured in a humid chamber for 28 days. Therefore, the obtained classes are underestimated and have to be considered as just a reference for contextualizing the materials with WM, indicating that the durability parameters are maintained at quite reasonable levels. In fact, increasing to 5% of WM could be equivalent to making a cement paste of w/c ratio 0.6 instead of 0.4.

Finally, it is important to make some considerations related to the upscaling of the technology. This paper intends to prove the suitability of including the masks in cementitious materials. In order to scale up the technology, it has to be considered that even, in this work, metal pieces and elastic bands were removed, they could be included, as it is reasonable to expect they will not have a deleterious effect on the resulting material. The optimum granulometry and the industrial procedure to shred the whole masks have to be optimized. In addition, used masks should be thrown away in specific containers. Thus, prior their manipulation, they have to be disinfected. There are several methods for this [42,47,48,49], and one of them will have to be implemented on a large scale. The selection of the most appropriate disinfection method is beyond the scope of this paper. Thus, this technology to reuse face mask waste is completely scalable. More work is being carried out in order to cover these upscaling aspects.

## 4. Conclusions

Adding MW to cast cement mortars supposes a decrease in the properties of the material, concerning both strength and durability behaviors. However, even adding 5% of WM in the weight of the cement, the aspect of the mortars is good, the flexural strength is not significantly affected, the percentage of loss of compressive strength after heating is similar to that of the reference, and the durability parameters are maintained at levels which are quite reasonable for use.

## Figures and Tables

**Table 1:** Mixes cast with different amounts of mask waste (MW).

	Cement I 52.5 R	Water	Sand	Superplasticiser (Master Ease 3690)	% MW Weight vs. Cem
MW-0	1	0.4	1	0.0065	0
MW-0.5-A	1	0.4	1	0.01	0.52
MW-1-A	1	0.4	1	0.01	1.0
MW-1-B	1	0.4	1	0.01	1.0
MW-2.7-A	1	0.4	1	0.01	2.66
MW-5-A	1	0.4	1	0.01	4.98

A—dry mixing of MW; B—MW mixed previously with 2/3 of the superplasticiser.

**Table 2:** Microstructure characteristics of samples.

	Total Porosity (%vol)	Bulk Density (gr/mL)	Average Pore Diameter (µm)
MW-0	9.58	2.19	0.033
MW-0.5-A	12.30	2.17	0.060
MW-1-A	14.46	1.99	0.044
MW-1-B	17.03	1.90	0.058
MW-2.7-A	15.73	1.94	0.058
MW-5-A	16.68	1.83	0.056

**Table 3:** Chloride diffusion coefficients (10^−12^ m^2^/s).

	D_eff_ Calculated from Resistivity [45]	D_eff_ Calculated from the Multiregime Method [44]	D_app_ Calculated from the Multiregime Method [44]
MW-0	2.54	—	—
MW-0.5-A	2.78	—	—
MW-1-A	5.61	3.06	5.50
MW-1-B	5.05	2.50	6.23
MW-2.7-A	4.52	2.53	5.64
MW-5-A	10.8	6.87	16.2

**Table 4:** Potential durability of the different mixes in this research according to the limits given in [46].

INDICATOR *	MW-0	MW-0.5-A	MW-1-A	MW-1-B	MW-2.7-A	MW-5-A
Total porosity (MIP)	Moderate	Moderate	Low	Very low	Low	Very low
Electrical resistivity	Low	Low	Very low	Very low	Very low	Very low
Effective Cl diffusion coefficient-D_eff_	—	—	Low	Low	Low	Low
Apparent Cl diffusion coefficient-D_app_	—	—	Moderate	Moderate	Moderate	Low

* limit values used prescribed for concrete.

## Data Availability

Not applicable.
